# Reviewer acknowledgement 2013

**DOI:** 10.1186/2050-7283-2-2

**Published:** 2014-02-10

**Authors:** Alice K Murray

**Affiliations:** BioMed Central, 236 Gray’s Inn, Road, London, WC1X 8HB UK

## Abstract

The editors of BMC Psychology would like to thank all our reviewers who have contributed to the journal in Volume 1 (2013).

**Neil Aggarwal**

USA

**Katie Alcock**

United Kingdom

**Steve Amireault**

Canada

**Glynne Andrew**

United Kingdom

**Katherine Applegate**

USA

**Amanda Baker**

Australia

**Steven Barger**

USA

**Jürgen Barth**

Switzerland

**Raquel Benbunan-Fich**

USA

**Dorothy Bishop**

United Kingdom

**Colin Bosma**

USA

**Kate Brain**

United Kingdom

**Adam Brickman**

USA

**Barbara Briers**

Netherlands

**Louisa-Jane Burton**

United Kingdom

**William Campbell**

USA

**Andreas Carlborg**

Sweden

**Julia Carroll**

United Kingdom

**Thep Chalermchai**

Thailand

**Tianyong Chen**

China

**Wenfeng Chen**

China

**Kaj Sparle Christensen**

Denmark

**Mark Conner**

United Kingdom

**Deirdre Conroy**

USA

**Silla Consoli**

France

**Philippe Conus**

Switzerland

**Richard Cooke**

United Kingdom

**Fiammetta Cosci**

Italy

**Patricia Dobkin**

Canada

**Carrie Donoho**

USA

**Janine Dretzke**

United Kingdom

**Angela Duckworth**

USA

**Andrew Dunn**

United Kingdom

**Thomas Ehring**

Germany

**Thomas Ehring**

Netherlands

**Pete Ellis**

New Zealand

**Ingegerd Ericsson**

Sweden

**Monica Eriksson**

Sweden

**Verena Ertl**

Germany

**Cláudia Ferreira**

Portugal

**Dara Friedman-Wheeler**

USA

**David Gallardo-Pujol**

Spain

**Bruno Giordani**

USA

**Melissa Gladstone**

United Kingdom

**Michael Gradisar**

Australia

**Florentia Hadjiefthyvoulou**

United Kingdom

**Jaana Halonen**

Finland

**Mechthild Hartmann**

Germany

**Phillipa Hay**

Australia

**Morten Hesse**

Denmark

**Nadja Heym**

United Kingdom

**Michael Horner**

USA

**Aimee James**

USA

**Peter Jonason**

Australia

**John Kasckow**

USA

**Alexia Katsarou**

Greece

**Maryanna Klatt**

USA

**Diana Kornbrot**

United Kingdom

**Banu Kuran**

Turkey

**Marius Lahti**

Finland

**Anna Law**

United Kingdom

**Erwin Lemche**

Germany

**Francois-Xavier Lesage**

France

**Christina Lessov-Schlaggar**

USA

**Doris Leung**

Hong Kong

**Dina Logiudice**

Australia

**Luigi Lombardi**

Italy

**Anna Macklin**

United Kingdom

**Leandro Malloy-Diniz**

Brazil

**Antonio Mantovani**

USA

**Chantal Martin-Soelch**

Switzerland

**Danielle Matthews**

United Kingdom

**Dana McMakin**

USA

**David Mellor**

Australia

**Laura Monetta**

Canada

**Erin Morgan**

USA

**Sharon Naismith**

Australia

**Sam Norton**

United Kingdom

**Russell Noyes**

USA

**Michael Obermeier**

Germany

**Thomas Olino**

USA

**Kimberly Oman**

Australia

**Vasiliki Orgeta**

United Kingdom

**Francesco Pagnini**

Italy

**Ståle Pallesen**

Norway

**Jan Patricia Piek**

Australia

**Karen Pine**

United Kingdom

**Stuart Ritchie**

United Kingdom

**Reuben Robbins**

USA

**Vera Santos**

Brazil

**Louis Schmidt**

Canada

**Marina Schoemaker**

Netherlands

**Beate M. Schulze**

Switzerland

**Louise Sharpe**

Australia

**Leigh Ann Simmons**

USA

**Shameran Slewa-Younan**

Australia

**Frank Snoek**

Netherlands

**Liliana Sousa**

Portugal

**Nicolas Stefaniak**

France

**Bradley Stein**

USA

**Anne Stephens**

Australia

**Randy Stinchfield**

USA

**Gijsbert Stoet**

United Kingdom

**Claire Taylor**

United Kingdom

**Jenna Todd Jones**

United Kingdom

**Teresa Torralva**

Argentina

**Richard Trigg**

United Kingdom

**Jane Ussher**

Australia

**Peter Van Der Velden**

Netherlands Antilles

**Charles Van Wijk**

South Africa

**Anouk Vanden Bogaerde**

Belgium

**Sargoor Veena**

India

**Tracey Wade**

Australia

**Jeremiah Weinstock**

USA

**William Wieczorek**

USA

**Matthias Wieser**

Germany

**Richard Williams**

United Kingdom

**Shannon Wiltsey Stirman**

USA

**Daniel Wiswede**

Germany

**Tingzhong Yang**

China

**Stefano Zago**

Italy

**Deborah Zaitchik**

USA

